# Powered wheelchair simulator development: implementing combined navigation-reaching tasks with a 3D hand motion controller

**DOI:** 10.1186/s12984-016-0112-2

**Published:** 2016-01-19

**Authors:** Gordon Tao, Philippe S. Archambault

**Affiliations:** School of Physical and Occupational Therapy, McGill University, 3654 prom Sir-William-Osler, Montréal, QC H3G 1Y5 Canada; Interdisciplinary Research Center in Rehabilitation (CRIR), Hôpital Juif de Réadaptation, 3205 Place Alton Goldbloom, Laval, QC H7V 1R2 Canada

**Keywords:** Virtual reality, Powered wheelchair, Training, Reaching, Navigation, Rehabilitation

## Abstract

**Background:**

Powered wheelchair (PW) training involving combined navigation and reaching is often limited or unfeasible. Virtual reality (VR) simulators offer a feasible alternative for rehabilitation training either at home or in a clinical setting. This study evaluated a low-cost magnetic-based hand motion controller as an interface for reaching tasks within the McGill Immersive Wheelchair (miWe) simulator.

**Methods:**

Twelve experienced PW users performed three navigation-reaching tasks in the real world (RW) and in VR: working at a desk, using an elevator, and opening a door. The sense of presence in VR was assessed using the iGroup Presence Questionnaire (IPQ). We determined concordance of task performance in VR with that in the RW. A video task analysis was performed to analyse task behaviours.

**Results:**

Compared to previous miWe data, IPQ scores were greater in the involvement domain (*p* < 0.05). Task analysis showed most of navigation and reaching behaviours as having moderate to excellent (*K* > 0.4, Cohen’s Kappa) agreement between the two environments, but greater (*p* < 0.05) risk of collisions and reaching errors in VR. VR performance demonstrated longer (*p* < 0.05) task times and more discreet movements for the elevator and desk tasks but not the door task.

**Conclusions:**

Task performance showed poorer kinematic performance in VR than RW but similar strategies. Therefore, the reaching component represents a promising addition to the miWe training simulator, though some limitations must be addressed in future development.

## Background

Reaching for objects, along with manoeuvrability within confined spaces, is a key factor in powered wheelchair (PW) mobility, i.e. the ability to overcome the physical and social obstacles of daily activities [1]. The ability to reach is necessary in a wide variety of common tasks: preparing food, working at an office, etc. Considering that PW users typically spend all their waking hours in their wheelchair [2, 3], learning how to best navigate their PW in order to reach for objects is crucial to their independence and quality of life. This advanced task-related training is often not possible in rehabilitation centers as access to training for PWs is already limited [4, 5].

Virtual reality (VR) simulators offer a highly feasible supplement for rehabilitation training either at home or in a clinical setting. Several simulators for PW are already in development [6–8]. Critical to the effectiveness of VR training is the transfer of skills to real-world scenarios [9–11]. To simulate reaching tasks for training, motion capture technology may be used to recreate the user’s manual movements in the VR environment, in real-time. In VR research, however, this usually requires large and expensive 3D cameras that are impractical for an at-home simulator [12–14]. Recent advancements in consumer-level motion-capture technology provide a low-cost and portable substitute. The focus of this study was to validate a six degree-of-freedom hand motion controller (Razer Hydra, Sixense, USA), as a training tool for reaching tasks in PW use. The Razer Hydra is a device that fits into one hand, similar in shape to a TV remote. The motion capture aspect of this device allows the user to physically control a virtual hand or cursor in 3D space with hand and arm movements. We chose this device for its low cost ($150 USD), portability, and ease of use (plug & play USB connection). This 3D hand motion controller was evaluated as an integrated tool in the miWe simulator.

An important consideration in VR research is the user’s sense of presence (SOP), i.e. the temporary suspension of disbelief such that users feel as if they were ‘in’ the VR environment [11]. Accordingly, presence is thought to make tasks in the VR environment feel more natural and relevant to the user and therefore could enhance task training and transfer of task performance to the real world (RW) environment. The SOP can be enhanced through visual immersion in the virtual world via head mounted display or large projection screens [8]. However, a greater degree of interactivity or the number of things that can be authentically *performed* in the virtual environment can also contribute to a greater SOP [15, 16]. Therefore, the addition of a 3D hand motion controller is expected to contribute to a greater SOP by providing a means of interacting with the VR environment using one’s hands.

The Hydra motion controller was integrated into the McGill Immersive Wheelchair (miWe) simulator, a VR training simulator for PW users. The miWe is a first-person perspective environment that runs on an ordinary computer. The virtual PW is controlled using a common PW joystick modified to connect to the simulator via USB. It can be used at home and is designed to teach PW users navigation and obstacle avoidance skills in the outside community. Manual tasks, such as opening doors, in the miWe were originally accomplished by keystroke. This project enabled users to physically perform such manual tasks in combination with manoeuvring their wheelchair in the simulator.

The objectives of this study were to:

1. determine to what extent using a 3D hand motion controller with the miWe simulator increases the SOP of PW users compared to those who use the simulator without the hand motion controller, and
2. compare, in a group of PW users, the performance of combined navigation-reaching tasks in a PW simulator using, using the hand motion controller, to those same activities performed in real life,
3. expound the way in which participants feel the combined navigation-reaching tasks, in context of the simulator, are appropriate for PW training.

## Methods

This study was approved by the Institutional review board of the Interdisciplinary Research Center in Rehabilitation (CRIR), reference number CRIR-728-0412.

### Population and sample

This study focused on the participation of experienced PW users, with a minimum of 2 years’ experience, as they were most likely to have stable behaviour and know how best to perform tasks; they were presumed to be familiar with scenarios encountered during activities of daily living. These users could provide expert opinions and feedback on the overall learning utility of the added reaching feature to the simulator from a standpoint of retrospective experience.

Participants were recruited from the Lucie Bruneau Rehabilitation Center (Canada). Participants needed to have a standard indoor/outdoor PW controlled by joystick. Other inclusion criteria consisted of: able to fully understand the tasks with a score of 13 or more on the Montreal Cognitive Assessment [17], have one able arm and hand for controlling the wheelchair, able to grasp at 40 cm forward and laterally, and normal or corrected vision. Potential participants were asked if they were able to perform the three tasks without assistance and only those who affirmed they could were recruited. Participants were excluded if they were unable to answer questions in French or English.

#### Ethics, consent and permission

Participants provided informed consent to participate according to CRIR guidelines.

#### Consent to publish

Participants provided informed consent to have their collected data published.

### Procedures

**Fig. 1:**
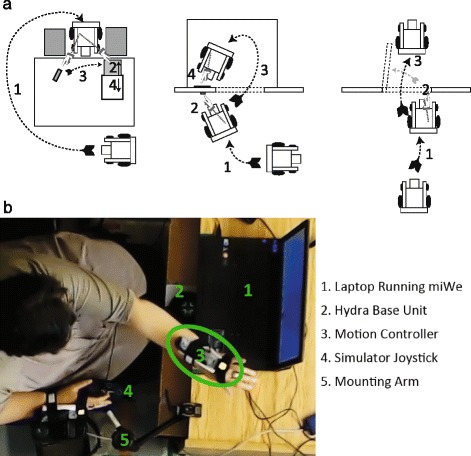
Task Diagrams (**a**). Illustrations of task progression for the ‘Desk’ (*left*), ‘Elevator’ (*middle*), and ‘Door’ (*right*). Simulator configuration (**b**)

Participants performed three tasks in the RW and in the miWe simulator (VR). These tasks were designed to reflect a variety of hand-arm movements (pushing, pulling, turning, transporting an object) with different levels of difficulty and are illustrated in Fig. 1a.

#### Desk

For this task, participants began in front of the desk. On the opposite side of the desk from where the participant began were two chairs with enough space in between to fit the PW. On the left side of the desk was a desktop drawer and on the right side was a lightweight circular object. Participants were tasked with manoeuvring around the table, parking between the two chairs, opening the drawer (pulling), placing the object in the drawer (contralateral reaching, object transport), and finally closing the drawer (pushing).

#### Elevator

Participants began diagonally away from the elevator’s door. They were tasked with manoeuvring into position to press the elevator’s call button (reaching forward or to the side), entering the elevator when the ‘door opened’ (6 s delay, indicated by auditory beep), and pressing the floor button (reaching forward or to the side).

#### Door

For this task, participants began directly in front and oriented towards the door. Participants were tasked to approach the door, open the door outward (hand-arm rotation, pushing), and proceed through the doorway. The task ended when the wheelchair crossed through the doorway.

#### Real-world and virtual environments

The starting environment was randomized, with 50 % of participants performing tasks in VR first. Participants performed each task 5 times in succession for each environment. After participants completed tasks in one environment, they proceeded to the second after a 5–10 min break. Instructions regarding how to perform the tasks in VR and RW were limited to the goals in the above task descriptions. Participants were encouraged to perform tasks ‘as they see fit’ and we avoided any instructions that suggested they perform tasks ‘as they normally would’ in VR.

The RW tasks took place in an open space facility with a table and doorway; for the elevator task, an elevator mock-up was used. Participants performed the RW tasks using their own PW, with maximal speed adjusted to their comfortable indoor speed. Since participants were compared to themselves, using their individually preferred speed was more accurate to their everyday performance than using a standard speed across participants. Participants were familiarized by verbal instruction and practice with each task, until they felt comfortable (approx. 15 min), before proceeding with data collection. The task order in RW was fixed to minimize time spent moving props and to keep total session time below three hours: *Elevator*, *Desk*, *Door*. Since these tasks are ordinary for experienced PW users, we reasoned that the order would minimally affect RW performance.

For VR, participants were seated in front of the computer monitor in their own PW (Fig. 1b). The user’s PW joystick was swivelled to the side and the simulator’s joystick was fixed, using an adjustable mounting arm, in the regular place of the PW joystick. The simulator joystick was similar to joysticks utilized by many PW models (Penny & Giles joystick, Traxsys, UK). The Hydra motion controllers (Sixense, USA) were modified with straps to be easily attached to the back of each hand. These are capable of one-to-one position and orientation tracking in 3D space. Each controller contains a magnetic sensor that interacts with a weak magnetic field produced by a base unit, providing tracking of the controller’s movement and orientation through space (accuracy of <1 mm and <1°). The advantage of this system is that it does not require line-of-sight between controller and sensor. Participants practiced navigating and interacting with objects in each virtual task (max 15 min).

For the VR tasks, environments developed for the miWe simulator included objects (doorway, desk, etc.) that were dimensional recreations of the objects used in RW; reaching and driving movements performed by the participant were translated 1:1 into the virtual world. The virtual PW was modeled as a generalized PW based on weight, acceleration, size, and directionality. We were interested in the reaching movement only and not in hand manipulations. Therefore, manual interaction in the simulator with objects was contextual and initiated by proximity, i.e. when the participant reached close enough to the object. For example, in the door task, the virtual hand would automatically grasp the door on proximity; however, the participant needed to supinate their forearm in order to rotate the handle 45°, then make a pushing gesture, which would cause the door to swing completely open.

In VR, participants generally performed the *Door* task first, followed by the *Desk* then *Elevator* tasks. We reasoned that while participants were given time to become familiar with the simulator, some learning effects were still likely to occur. Therefore, the *Door* task, as the most straight-forward task and least likely to see varied strategy, was presented first. However, the ultimate order depended on the participant; sometimes a participant had difficulty completing a given task at first—this task was revisited after completing the other tasks.

### Data collection

The iGroup Presence Questionnaire (IPQ) was used to determine the users’ SOP while using the miWe simulator. We used the IPQ to determine if the addition of the motion-controlled reaching interaction increased SOP for the miWe. The IPQ comprises of fourteen items in four subcategories: Spatial Presence, Involvement, Experienced Realism, and a general ‘sense of being there’ [18]. All items are scored on a seven-point scale (0–6), with a higher score indicating a greater SOP. The IPQ has good internal consistency, with with Cronbach’s alpha of 0.87 for the complete scale and approximately 0.75 for each subscore [19]. The IPQ was administered immediately after completing the all tasks in VR and RW.

For joystick position data in RW, we used a modified PW joystick connected to a data logging system [7], which sampled at 200Hz, that we mounted on the participant’s wheelchair. We also used a ceiling-mounted wide-lens Logitech HD Pro Webcam C920 (Morges, Switzerland) with an average sampling rate of 30 Hz at 1080p resolution and encoded in H.264/MPEG-4 to record video data.

For VR, the miWe simulator recorded joystick input, virtual PW position, and motion controller position and orientation at an average sampling rate of 50 Hz, corresponding to the simulator’s video frame rate.

Participant-reported data was collected from a task-specific and general questionnaire that prompted participants to provide feedback regarding usability, relevance, limitations, improvements, etc. for the simulator. The questionnaire was similar to the feedback form used in a previous miWe study [20]. We emphasized feedback regarding the reaching component and the motion controller. The feedback form was administered immediately after the IPQ.

### Analysis

IPQ scores were compared to data gathered from a previous study on the miWe without the reaching component using an independent t-test [20]. Questionnaire data were summarized with common trends.

Video and joystick data from the RW environment were time synchronized by the manual marking of the onset of first joystick movement—the task start. Task completion time was determined by task-specific criteria: closing the drawer, pressing the floor button of the elevator, and crossing through the doorway. Furthermore, time spent reaching was defined by the onset of a reaching movement towards a reaching objective to the end of returning the arm to rest or the end of task completion criteria.

In both the RW and VR environments, joystick excursion was calculated via the vector norm of x and y displacements. Joystick movements were defined as an excursion away from the neutral position (threshold of 10 %). Since the wheelchair brakes automatically engaged when the joystick was in neutral position, this definition provided a clear picture of go-stop driving patterns.

Number of reaching movements was counted by visual inspection of video data for RW tasks and from the hand position data collected from the simulator for VR (cross referenced with video data). A reaching movement was defined as ‘an effortful movement towards an object’; thus, pressing a button counted as 2 movements (reach to press and return to rest), while reaching to grasp with lateral drifting (searching behaviour) counted as one movement.

Quantitative measures were compared across participants in pairwise fashion. Data pairs (RW, VR) were the mean of the five trials per task per environment. Wilcoxon sign-rank tests were used to compare non-normally distributed continuous and count data and t-test was used for normally distributed data.

A video task analysis was performed to compare task errors and behaviours. For each task, two unblinded researchers identified and agreed on *sub-tasks* that comprised of a single goal, e.g. driving up to the door. Within each sub-task, two researchers independently identified (across all trials) on distinct *behaviours* that could vary from trial to trial. Any disagreement on these observations was solved by discussion until a consensus was reached. Each *behaviour* comprised of mutually exclusive options and both researchers agreed on strict contextual criteria for coding. One researcher coded all trials and a third blinded researcher verified the coding of 3 participants for consistency. Task behaviours included strategies (e.g. which hand was used for reaching) and performance characteristics (e.g. un/interrupted driving). Errors were evaluated similarly, but separately from task behaviours using relative risk assessment.

Concordance between RW and VR tasks was determined for each behaviour of each task. For a given behaviour, each participant’s dominant behaviour in RW was identified and compared to their corresponding dominant behaviour in VR. To illustrate, we may consider the behaviour, ‘drive forward or reverse into the elevator’, during the *Elevator* task. If the participant reversed in 5/5 times in RW and 3/5 times in VR, the dominant behaviour in both environments would be ‘reverse’. Therefore, behaviour, ‘forward/reverse in’, would be rated as concordant for the *Elevator* task of the participant. If, however, the participant reversed in 3/5 times in RW and 2/5 times in VR, the dominant behaviour in RW would be ‘reverse’ and the dominant behaviour in VR would be ‘forward’. Therefore, the dominant behaviour would be rated as discordant between the two environments.

Each behaviour for each participant was rated concordant/discordant (1/0); ‘dominant behaviour’ in this context was selected to describe the expert participants’ preferred behaviour and most likely reflects their ‘best practice’ for task performance in RW. We wanted to see how closely VR performance matched these behaviours. Furthermore, PW users do not always perform tasks in the same manner in RW. A VR simulator that elicits authentic behaviour should also reflect this.

Concordance of task behaviours across participants was determined using Cohen’s Kappa coefficient:

$$ K=\frac{ \Pr (a) - \Pr (e)}{1- Pr(e)}, $$

where Pr(*a*) is the proportion of counted concordant cases and Pr(*e*) is the proportion of concordant cases due to ‘random chance’ behaviour, e.g. the Pr(*e*) of ‘used hand’ would be 0.5; for the purposes of this study, we assumed equal proportion of all identified behaviours for Pr(e). Kappa values were evaluated according to guidelines proposed by Fleiss [21].

## Results

Of fourteen people recruited, four women and eight men aged between 36 and 60 years (50.1 SD 9.1) participated in this study (Table 1). One person proved unable to perform our tasks independently on arrival and did not participate and another failed to appear due to prior injury. The participants had between 2 and 30 years (16.1 SD 9.7) experience using their PWs and an overall MOCA score of 20.1 SD 4.5. The right-handed to left-handed ratio was 1:1.

**Table 1:** Participant descriptions

Participant	Age	Sex	Experience	Handed	PWC	MoCA
1	36	M	24	R	Rear-Wheel	15
2	60	F	14	L	Rear-Wheel	19
3	51	F	23	L	Rear-Wheel	13
4	55	F	10	L	Rear-Wheel	25
5	51	M	20	L	Mid-Wheel	20
6	56	M	30	L	Mid-Wheel	18
7	56	F	2	R	Rear-Wheel	25
8	37	M	25	R	Rear-Wheel	24
9	59	M	4	R	Rear-Wheel	26
10	59	M	26	L	Rear-Wheel	21
11	38	M	10	R	Mid-Wheel	14
12	43	M	5	R	Rear-Wheel	21

Age (years), Sex (Male, Female), Experience (years), Handedness (Right, Left), MoCA = Montréal Cognitive Assessment Test (max = 30)

All participants completed all tasks for both RW and VR environments. In VR, 10 of 12 participants completed the door task first. Two participants needed to complete the session over 2 days, with one environment (VR/RW) completed on each day. In both cases, the session was delayed by difficulties adapting the PW joystick logger to the participant’s PW.

Measures of task completion time, reaching time, joystick movement count, and reaching movement count were averaged for each task for each participant. Distributions of these averages were found to be non-normal (Shapiro-Wilk, *p* < 0.05). Therefore, the Wilcoxon signed-rank test was appropriate for comparing paired data.

### Presence

**Fig. 2:**
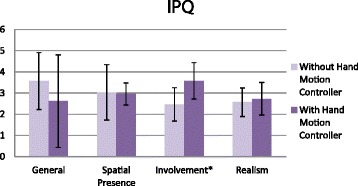
iGroup Presence Questionnaire. IPQ scores are summarized across 4 domains compare previously gathered miWe data without the 3D hand motion controller and our results. Error bars represent standard deviation and (*) is significant (*p* < 0.05)

Presence was measured for the VR simulator using the IPQ; scores were normally distributed and are summarized with mean and standard deviation in Fig. 2. Overall, ‘general sense of presence’, ‘spatial presence’, and ‘realism’ averaged slightly below three; only ‘involvement’ averaged above three. In comparison with previous data collected on the miWe [20], without a reaching component, our results showed a significantly greater (independent t-test, *p* < 0.05) sense of ‘involvement’. However, scores in the remaining three categories were not significantly different.

### Task times

The *Elevator* task took the longest total time with a median time of 24.9 s (interquartile range [IQR] 21.4—31.7 s) in RW and 57.6 s (IQR 42.6—69.2 s) in VR. The median time for the *Desk* task was 19.5 s (IQR 15.3—24.9 s) in RW and 52.6 s (IQR 39.4—77.4 s) in VR. The *Door* task took the least time overall with a median task time of 10.6 s (IQR 7.5—14.4 s) in RW and 13.4 s (IQR 42.6—69.2 s) in VR.

**Fig. 3:**
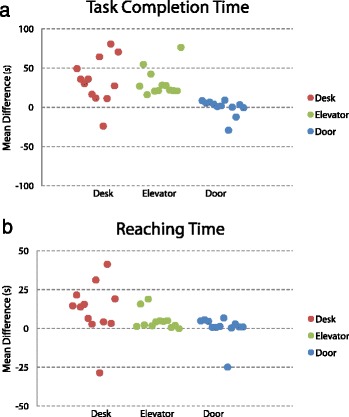
Task Time Mean Difference. **a** shows differences in total task time and (**b**) shows differences in time spent reaching. Values > 0 indicate greater time in VR compared to RW. Each point represents the mean difference between environments for one participant

Only the *Door* task demonstrated similar task completion times with no significant difference between VR/RW (*p* = 0.3). For the *Desk* and *Elevator* tasks, the VR completion times were both significantly (*p* < 0.01) longer; the *Desk* task showed the greatest difference: median 33.0 s (IQR 15.4—53 s) longer to complete in VR than RW. A plot of task completion time differences is shown in Fig. 3a.

Within each task in the RW, participants spent time reaching for objects a median of 6.1 s (IQR 4.8—9.0 s) in the *Desk* task, 5.2 s (IQR 4.1—6.7 s) in the *Elevator* task, and 3.8 s (IQR 3.4—5.6 s) in the *Door* task. By comparison, participants spent significantly more time in VR for all three tasks (*p* = 0.02 for *Desk*, *p* < 0.01 for *Elevator*, *p* = 0.03 for *Door*). The greatest difference in reaching time was in the *Desk* task where participants took a median 14.2 s (IQR 4.0—19.7 s) longer for reaching in VR than in RW. A plot of reaching time differences is shown in Fig. 3b.

### Discrete movements

**Fig. 4:**
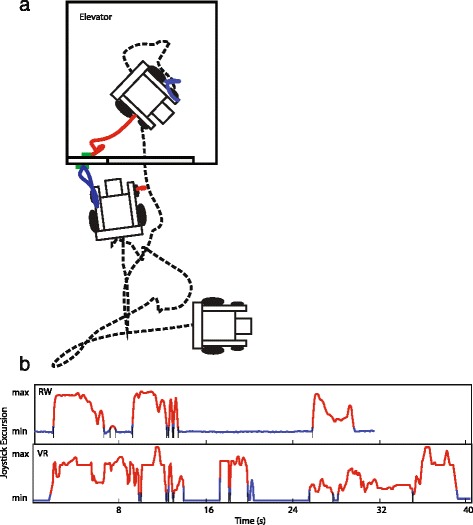
Elevator Trial Examples. **a** show the position traces, in a VR trial, of the wheelchair (*black*) and hands during reaching in *red* and *blue*. **b** shows examples of joystick excursion during elevator trials in RW and VR; discrete ‘joystick movements’ are highlighted in red

Figure 4a shows an example of the *Elevator* task performed by one participant in VR. Figure 4b shows example traces representing joystick excursion during the *Elevator* task in RW and in VR. The complexity of the navigation component of this task is demonstrated by the number of joystick movements. Moreover, the VR trace shows noticeably more joystick movements than in the RW counterpart.

**Fig. 5:**
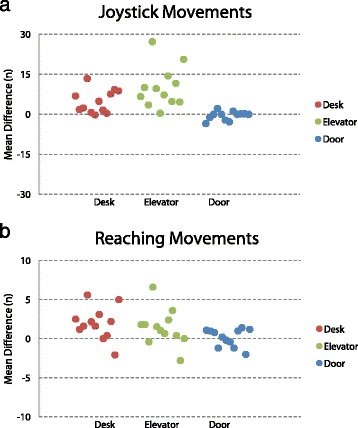
Movement Count Mean Difference. **a** shows differences in total joystick movements and (**b**) shows differences in total reaching movements. Values > 0 indicate greater time in VR compared to RW. Each point represents the mean difference between environments for one participant

Within each task in the RW, participants completed tasks with a median number of joystick movements of 2.7 (IQR 1.8—4.5) in the *Desk* task, 4.4 (IQR 3.4—6.0) in the *Elevator* task, and 3.6 (IQR 2.4—5.2) in the *Door* task. By comparison, participants utilized significantly more joystick movements in VR for the *Desk* task (*p* < 0.01) and *Elevator* task (*p* < 0.01). The greatest difference was in the *Elevator* task where the VR environment required a median of 8.4 (IQR 4.8—12.3) more joystick movements than in RW. However, joystick movement count was concordant between RW and VR in the *Door* task, with no significant difference between counts (*p* = 0.45). A plot of joystick movement differences is shown in Fig. 5a.

Similarly, the number of reaching movements utilized was concordant between RW and VR only for the *Door* task (*p* = 0.74). A significantly greater number of reaching movements were made in the VR environment for the *Desk* task (*p* < 0.01) and *Elevator* task (*p* = 0.04). The greatest difference was in the *Desk* task where the VR environment required a median of 1.9 (IQR 1.0—2.7) more reaching movements than in RW. For comparison, participants required a median count in RW of 6.3 (IQR 6.0—6.7) in the *Desk* task, 3.8 (IQR 3.4—4.1) in the *Elevator* task, and 4.1 (IQR 3.8—4.7) in the *Door* task. A plot of reaching movement differences is shown in Fig. 4b.

### Task analysis

Two task errors and twenty-two behaviours were identified throughout all sub-tasks . The task errors were collisions (driving error) and errors in judgment of maximum reaching distance (reaching error). A collision was defined as any contact of the PW with an obstacle. An error in reaching distance occurred when the participant misjudged the distance required to reach a target (e.g. a button) and needed to adjust their PW position closer. Trials were divided into those where one or more driving errors occurred and those where no collisions occurred; the same procedure was done for reaching errors. For each task and across all participants, there was a significantly greater relative risk (RR, *p* < 0.05) of errors occurring during a trial performed in VR than in RW across all tasks: *Desk* (RR driving = 3.72, RR reaching = 2.93), *Elevator* (4.31, 5.86), *Door* (1.89, 1.5).

Table 2 describes all the sub-tasks identified and Table 3 describes all of the behaviours identified for each sub-task. These were developed from the task analysis for this study.

**Table 2:** Sub-tasks. Tasks are broken down into sub-tasks and progress chronologically. Task behaviours are organized into each component. Some behaviours appear in multiple sub-tasks of the same task

Desk	Elevator	Door
Navigating around desk	Navigate to button 1	Advancing towards door
DG1, DG3, RG1	DG1, DG3, DEl1, RG1	DG1, DG3, DDr1, RG1
Parking between chairs	Parking at button 1	Parking in front of door
DG2, DG3, RG3	DG2, DG3, RG3	DG2, DG3, RG3
Reaching for object	Pressing button 1	Reaching for door handle
RG2, RDe1, RDe2	RG2	RG2
Opening drawer	Navigate to button 2	Opening door
RG2, RDe2	DG1, DG2, DG3, DEl2, DEl3, DEl4, RG1	RDo1
Placing object in and closing drawer	Parking at button 2	Driving through doorway
RDe2, RDe3	DG2, DG3, RG3	RDo2
	Pressing button 2	
	RG2

**Table 3:** Task Behaviours. Behaviours are described in detail and given a summary code. Codes with ‘G’ are general behaviours observable in all tasks

Code	Driving Behaviour	Code	Reaching Behaviour
DG1	Fluid drive-to-park: participants completed navigation without pausing	RG1	Start reaching before park: participants began reaching before their PW completely stopped
DG2	Parking Position: 2–4 positions depending on context	RG2	L/R hand: which hand was used to reach the target
DG3	Collision: any contact of the wheelchair with an obstacle	RG3	Adjust parking close for reach: misjudged reaching distance, i.e. reaching error
DEl1	Turn first/reverse first: how participants began navigating to the first button	RDe1	Pickup order: the object was picked up before, after, or at the same time as opening the drawer
DEl2	Waiting for door: participants waited for the door without repositioning their PW	RDe2	Heavy leaning: participants needed clearly uncomfortable trunk compensation to reach target
DEl3	Forward/reverse in: entering the elevator	RDe3	Close drawer hand: participants closed the drawer with either the same or opposite hand that placed the object inside the drawer
DEl4	Horizontal Adjust: a characteristic ‘S’ manoeuvre sideways	RDo1	Fluid turn + push: the door was opened in a single, non-segmented movement
DDo1	Advance straight: participants drove in a straight line to the door	RDo2	Hand still raised: while driving through the doorway

Letter Codes: Driving (D), Reaching (R), General (G), Elevator (El), Desk (De), Door (Do)

Performance in the *Desk* task showed fair (0.4 < *K* < 0.75) to excellent (*K* > 0.75) agreement for most reaching behaviours except for the ‘close drawer hand’. However, driving behaviours showed poor agreement. Of note, the ‘fluid drive-to-park’ behaviour showed a negative Kappa score (*K* = −0.17), indicating that participants tended towards the opposite behaviour in VR. Performance in the *Elevator* task showed generally fair agreement between RW and VR for driving and reaching behaviours. Only the ‘fluid drive-to-park’ and ‘waiting for door’ behaviours showed poor agreement. Finally, performance in the *Door* task showed generally fair agreement for driving and reaching behaviours except for the ‘advance straight’ and ‘fluid turn and push’ behaviours. All agreement statistics are listed in Table 4.

**Table 4:** Task Behaviours Agreement. Summary of concordance, as measured by Cohen’s Kappa coefficient (K), for each characteristic in each task across participants

		K
Desk		
Driving Behaviour	Fluid drive-to-park	−0.17
	Parking Position	0.33
Reaching Behaviour	Start reaching before park	0.83
	Pickup order	0.50
	Close drawer hand	0.20
	L/R Hand	0.80
	Heavy leaning	0.67
Elevator		
Driving Behaviour	Turn First/Reverse First	0.83
	Fluid drive-to-park	0.22
	Parking Position	0.45
	Waiting for Door	0.17
	Forward/Reverse In	0.67
	Horizontal adjust	0.67
Reaching Behaviour	Start reaching before park	0.67
	L/R Hand	0.60
Door		
Driving Behaviour	Advance straight	0.17
	Fluid drive-to-park	0.67
	Parking Position	0.67
Reaching Behaviour	Start reaching before park	0.50
	L/R Hand	0.80
	Fluid turn + push	0.33
	Hand still raised	0.67

### Questionnaire and feedback

**Fig. 6:**
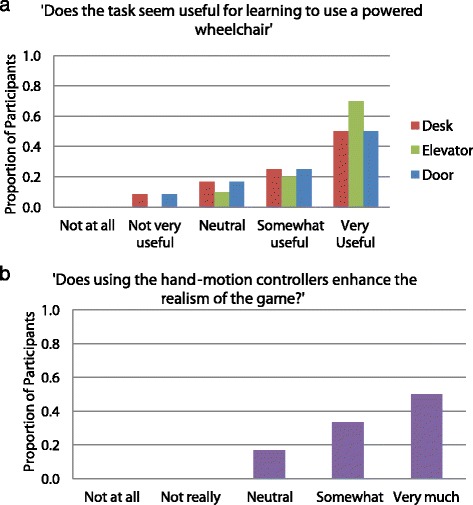
Example questionnaire items, task specific **(a)** and general **(b)**, and participants’ responses

Participants answered additional task specific and general questions (Fig. 6) on a 5-point Likert scale (Not at all, Not very, Neutral, Somewhat, Very) and were prompted to make task specific and general suggestions and comments (Table 5) about the VR simulator.

When asked, ‘Does the task seem useful for learning to use a powered wheelchair’ in context of the VR simulator, 9/12 participants or more rated each of the tasks as somewhat or very useful. When asked, ‘Does this task seem realistic’, nearly all (10/12 or more) participants rated tasks as somewhat to very realistic. However, only 6/12 to 7/12 participants rated the difficulty of the simulator tasks as somewhat to very appropriate. Likewise, 5/12 to 6/12 participants rated the tasks as somewhat to very motivating. Finally, 10/12 participants rated the added realism due to the Hydra as somewhat to very much (Fig. 6b).

Participants’ comments regarding specific tasks and the simulator in general varied and included both positive and negative feedback. One participant commented, ‘Interesting. I like the word “success” in the end, it's motivating!’ Another participant commented, ‘I was a little stressed. I do not like playing with joysticks in a virtual environment’. Participants were also prompted to suggest other relevant tasks; one task was suggested more than once: ‘getting coffee’. A list of common comments is summarized in Table 5.

**Table 5:** Questionnaire Feedback Comments. Summary of the most frequent comments. Comments were generalized to give an overall impression of how participants responded. Categories represent topical prompts to which participants responded

Category	Generalized Comment	Count
Criticism of the tasks	Difficulty with joystick precision and accuracy	5
	Lacking of vision due to fixed camera	3
	Elevator was too small	3
Reactions to the reaching controller	Enjoyed using the reaching controller	5
	Noted ‘glitches’ during reaching tasks	3
	‘It adds realism to the game’	2
	Difficulty due to lack of depth perception	2
Suggestions	Changes to the task that would add complexity	5
	Changes to the task that would lower complexity	5
	Specified other indoor tasks	6
	Specified other outdoor tasks	1

## Discussion

We anticipated that the inclusion of the navigate-to-reach component and the Razer Hydra 3D hand motion controller interface would increase the SOP in the miWe simulator. In the previous miWe study, where manual tasks were performed by keystroke, average scores were above three for general sense of presence and spatial presence, but below three for involvement and realism [20]. In our study, involvement showed a significant increase in the involvement score; this likely reflects a positive effect from the added interactivity provided by the hand controllers and the increased complexity, both physically and cognitively, of combined navigation-reaching tasks. Interestingly, no significant changes were observed in the other categories. We believe that drawbacks in the implementation of the virtual environment and simulation may have counteracted potential gains from the implementation of the hand controller and reaching tasks in the general, spatial presence, and realism categories of the IPQ. Examination of participant feedback, task performance, and task strategies help elucidate how this is the case.

### Participant feedback

Participants responded positively with respect to training utility, realism, and graphical quality. The task difficulty and motivation were judged less positively, but were generally neutral or better. These results suggest that the overall design of the tasks and task environments were representative of tasks in RW. Participants’ comments also reflected frustration with the some aspects of the simulator; they indicated that VR tasks felt more difficult to accomplish than the corresponding RW tasks, particularly because controlling the PW was harder and noticeably different from what they were used to in RW. A previous study comparing PW driving in VR vs RW showed similar reactions from participants [22]. However, several participants also commented that the reaching tasks were fun and engaging, indicating the potential for improving the motivational aspect of the simulator.

Overall, the feedback comments were ambivalent, with some participants responding very positively and others having severe difficulty with the simulator. One factor to consider in these responses is the novelty of the PW simulator, particularly with respect to the reaching component; since the study was cross-sectional, the novelty of the situation may have led to greater initial enthusiasm for those who already enjoyed VR experiences (e.g. videogames) and insufficient familiarization for those who were already anxious about VR environments. Furthermore, these data were from PW users recruited from one site in Montréal, which may limit generalizability. On the other hand, participants represented ages spanning a range of 23 years and experience ranging from 2 to 30 years; they were also providing feedback with respect to fairly rudimentary and universal tasks. In context of the participants’ SOP, their feedback regarding realism of task presentation reflects positively on their SOP. However, it seems the overall difficulty of performing the tasks in VR was likely a key factor in the lack of change in the IPQ realism score.

Participants also largely viewed the simulator as useful for learning to use a PW. It may have been some time since experienced users first learned to use their PW and may have forgotten what the specific challenges they had to go through. However, expert users will have likely experienced a wider range of situations and challenges, compared to beginners and therefore would have a more complete perspective on the learning utility of the simulator.

### Task performance

We compared task performance between RW and VR environments. We found that, in two of three tasks (*Desk* and *Elevator*), task completion time, time spent reaching, number of joystick movements, and number of reaching movements were all significantly greater when the task was performed in VR compared to RW. Only the *Door* task showed any concordant performance measures.

Joystick movements were represented by continuous deviation from the joystick neutral position and therefore reflect distinct PW manoeuvres. As such, concordant joystick movements in the *Door* task indicate that participants followed similar manoeuvring sequences in VR and PW; discordant joystick movements for the *Desk* and *Elevator* tasks indicate that participants navigated to objectives differently.

Few studies have directly compared PW task performance between VR and RW. Similar to our results, the study by Harrison and colleagues [22] reported greater task completion times and a greater number of discrete manoeuvres in VR compared to RW when comparing several manoeuvring tasks and one route-finding task. However, the previous study involving the miWe simulator [20] showed no significant difference in task completion times in 4/7 Wheelchair Skills Test [23] type tasks and no significant difference in the number of joystick movements in 6/7 of these tasks. The authors noted that the tasks with significantly greater times and movements were the most difficult ones. Specifically, in their ‘Door (Push)’ task, participants lined up with the door, ‘pushed’ the door open, drove through, turned around, and closed the door. In comparison, our *Door* task required no turning and minimal manoeuvring through tight spaces. Therefore, as our simplest task, it is unsurprising that the *Door* task was the one that did show concordant performance in total task time and joystick control.

Our video task analysis showed a higher risk of collisions in VR compared to RW. Of note, collisions occurred in 100 % of *Desk* trials and 93 % of *Elevator* trials in VR. Similarly, Harrison and colleagues [22] counted a total of 4 collisions over all their RW trials and 140 over all VR trials, in their manoeuvring and route-finding tasks. The authors noted that collision rates were greatest in tasks that required turning. Furthermore, Archambault and colleagues [20] noted frequent collisions and adjustments in VR compared to RW for their most complex manoeuvring tasks. This is consistent with our results where the greatest relative risk of collisions in VR were with regard to the *Desk* and *Elevator* tasks, which required turning in tight spaces. Therefore, corrective and repeat movements for collisions likely contributed to greater task times. The poor agreement of the driving behaviours ‘advance straight’ (*Door* task) and ‘fluid drive-to-park’ further suggests that navigation was overall more efficient in RW than in VR.

The poorer navigation performance is reflective of the participants’ feedback regarding difficulty of control and may be explained by a number of simulator limitations. The miWe simulator has a fixed field of view (FOV) of the virtual world; the virtual camera is fixed in the forward position and participants cannot easily ‘look around’ their wheelchair to focus on obstacles. Moreover, the miWe system uses a 2D, monoscopic display instead of a 3D stereoscopic display, meaning participants lacked depth perception for judging distances to obstacles. Participants also responded on the questionnaire that the joystick control of the PW was unrealistic, e.g. wide turn radius and needing improvement in precision and accuracy. Together, these limitations make obstacle avoidance, especially in tight spaces, more challenging in VR than in RW and likely make a major contribution to the observed discordance in navigation performance.

For cognitive skills such as path finding and overall task planning, however, participants tended to adopt concordant (moderate to excellent agreement) strategies when making navigation choices in VR and RW: they generally parked in the same position relative to the reaching target and they approached the buttons on the elevator in the same relative orientation. Also, the majority of observed reaching behaviours and strategies were concordant (moderate to excellent agreement) for each task in VR and RW: participants tended to use the same hand for reaching a given object and had similar start-of-reach timing relative to parking. This suggests that, while participants may have driven less efficiently in VR compared to RW, they still performed both route finding and task planning (for navigation and reaching components) in similar ways.

Reaching movements were represented by distinct arm movements to a target. Similar to the joystick results, participants showed concordant reaching sequences in the *Door* task and discordant reaching sequences in the *Desk* and *Elevator* tasks.

To our knowledge, this study is the first to compare reaching performance between VR and RW in context of using a PW. However, there is much research on motor performance in VR with respect to upper-limb rehabilitation [9]. Of note, Viau and colleagues [24] compared motor performance and movement patterns between VR (2D display) and RW during a reach-grasp-release task. These authors reported similar overall movement strategies between the two environments, but different movement with respect to degree of elbow and wrist extension. However, other studies have demonstrated mixed results [12, 25–27]. Some of the differences in performance in VR (both 2D and 3D) have been attributed to the presence or absence of haptic feedback [28] and display platforms [29].

The lack of haptic feedback and depth perception in our study likely contributed to the greater difficulty reported by participants and explain the greater number of reaching movements (*Door* and *Elevator* tasks) and reaching time (all tasks) observed in our study; one participant commented in the questionnaire that they had difficulty knowing if they had pressed the button in the *Elevator* task, even with the visual feedback (button lighting up). Additionally, the reaching tasks in our study differed from other studies in that the starting position of the user relative to the reaching objective was variable and dependent on the participant’s judgement; they decided where to park. As such, participants were required to accurately judge that the reaching objective was indeed within reach. Our results showed that, for all tasks, participants were more likely to misjudge this reaching distance in VR, i.e. higher relative risk of reaching errors.

Overall, the above mentioned limitations in the simulator may have contributed negatively to participants’ SOP and likely countered potential gains in the realism, spatial presence, and general sense of presence categories of the IPQ due to our implementation of the Hydra controller and combined navigation-reaching tasks. Unfortunately, it seems the greater complexity and number of features we have for a simulator, the more opportunities there are for participants to experience unrealism in some aspect of the simulator.

A potential solution for many of these issues may be the utilization of low cost consumer-level HMDs, e.g. the Oculus Rift (Oculus VR, Irvine, USA), featuring a 1080p resolution stereoscopic display, a physical 90° by 110° FOV, and head motion tracking capabilities. This would provide greater visual immersion, depth perception, and enable users to look around the virtual environment using head movements and may be implemented in future versions of the simulator.

Furthermore, the upcoming Sixense STEM system (Sixense, USA) is the next generation of the Hydra motion controller. The STEM system features small blocks that feature just the motion sensor and additional haptic feedback and could be readily implemented in the miWe. These blocks could more easily be attached to a user’s hands and the haptic feedback would increase the interactivity of reaching in the miWe.

## Conclusions

In summary, this study on the Razer Hydra 3D motion controller provides evidence supporting its use as an interface for combined navigation-reaching tasks in the miWe simulator. SOP was increased in the involvement domain compared to the simulator without reaching; participants demonstrated concordant task behaviours and strategies and concordant kinematic performance on the least difficult task between VR and RW; and feedback from participants indicated that the combined navigation-reaching tasks were appropriate, useful for PW training, and engaging. Therefore, this device is a valid interface for training and familiarizing combined manual-navigation tasks from a task planning/strategy standpoint and may be utilized in future versions of the simulator. However, important limitations of the simulator explain discordant measures and ultimately the lackluster increases in participants’ SOP; therefore, future development of the simulator must address these issues through improved hardware and refinement of the virtual environment.

We believe that, in more complex tasks, fundamental differences between VR (e.g. PW steering characteristics, depth perception, multiple interfaces) and RW are more likely to be compounded, resulting in divergent task performances. However, it is this very capacity for complex tasks that marks a key advantage of VR simulators. It allows us to expand traditional training to include, in VR, a greater variety of stakeholder-relevant tasks, scenarios, and difficulty levels. In context of this study, we may consider any situation, at home or in the community, in which PW users would need to interact using their hands, i.e. capitalizing on the integration of a 3D had motion controller. Therefore, future development will need focus on minimizing the experiential differences, with an emphasis on difficulty optimization, from the real world. Some of these developments in the miWe are underway and reported by Archambault and colleagues [30].

## Abbreviations

FOV: Field of View
IPQ: iGroup Presence Questionnaire
IQR: Interquartile Range
miWe: McGill Immersive Wheelchair Simulator
PW: Powered Wheelchair
RR: Relative Risk
SOP: Sense of Presence
VR: Virtual Reality
